# A circular RNA derived from DAB1 promotes cell proliferation and osteogenic differentiation of BMSCs via RBPJ/DAB1 axis

**DOI:** 10.1038/s41419-020-2572-3

**Published:** 2020-05-15

**Authors:** Weekai Chia, Jiali Liu, Yi-Gang Huang, Changqing Zhang

**Affiliations:** 1 Department of Orthopedics, Shanghai Jiao Tong University Affiliated Sixth People’s Hospital, No.600 Yishan Road, 200233 Xuhui District, Shanghai City, China; 2 Department of Oncology, Shanghai Jiao Tong University Affiliated Sixth People’s Hospital, No.600 Yishan Road, 200233 XuhuiDistrict, Shanghai City, China

**Keywords:** Biotechnology, Cancer, Cell biology

## Abstract

Osteogenesis (OS) is a type of differentiation that is of great importance for bone homeostasis. Increasing studies suggest circular RNAs (circRNAs) as pivotal regulators in OS. This study proposed to investigate mechanism mediated by circRNAs in OS. Based on GEO data and qRT-PCR assay, we found that circ-DAB1 (has_circ_0113689) was significantly up-regulated during osteogenic differentiation in human BMSCs. Overexpressing circ-DAB1 proliferation and osteogenic differentiation of BMSCs, whereas silencing circ-DAB1 elicited opposite functions. Subsequently, recombination signal-binding protein for immunoglobulin kappa J region (RBPJ), an important transcription factor in NOTCH pathway, was found to interact with DAB1 promoter while not to combine with circ-DAB1. Interestingly, circ-DAB1 overexpression could result in the increasing binding between RBPJ and DAB adaptor protein 1 (DAB1) promoter. Overexpressing circ-DAB1 upregulated RBPJ in BMSCs to induce DAB1 level. Further, we uncovered that circ-DAB1 upregulated RBPJ through sequestering miR-1270 and miR-944. Restoration experiments demonstrated that knocking down either RBPJ or DAB1 partially recovered BMSC proliferation and osteogenic differentiation that was suppressed by circ-DAB1 overexpression. Conclusively, circ-DAB1 promotes cell proliferation and osteogenic differentiation of BMSCs via NOTCH/RBPJ pathway.

## Introduction

For orthopedic and craniofacial surgeons, it is challenging to treat non-healing bone defects resulting from tumor ablation, trauma and infection. Several factors limit the efficacy of traditional bone grafting therapeutics with autologous bone, including donor site pain, low availability, and morbidity. The continuous remodeling helps maintain bone homeostasis, and this process mainly depends on the concerted balance between resorption and formation of osteoblastic bone^1,2^. Human bone marrow stem cells (BMSCs) are a type of multipotent progenitor cells with self-renewal property and diverse differentiation potential^3,4^. BMSCs provide critical source for bone formation, and can successfully enhance bone repair^5^. Importantly, the disrupted osteogenic process of BMSCs could break the balanced of bone homeostasis. Thus, understanding the regulatory mechanisms underlying osteoblast differentiation of BMSCs will contribute to developing potential therapeutic strategies. A large number of coding and noncoding RNAs are reported to effectively regulate the osteogenic differentiation of BMSCs^6,7^, and in this way, biomarkers and modulatory network have been identified. However, further elucidation of functions of noncoding RNA-related mechanism in OS is still required.

Recent studies highlight the significant role of circRNAs in regulating gene expression^8,9^. CircRNAs are loop-structured noncoding RNAs that are stable enough to resist RNase R digestion^10^. CircRNAs are formed by alternative splicing (back-splicing) and the connection between an upstream splice acceptor and a downstream splice donor^11,12^. Compared with long non-coding RNAs (lncRNAs) and microRNAs (miRNAs) in mammalian cells, circRNAs are more stable and conserved^13,14^. Functionally, circRNAs can affect expression of associated genes by acting as “miRNA sponges”, modulating transcription, mediating RNA processing reactions, and acting as scaffolds or templates for protein complex assembly or translation^15,16^. Existing reports identify several functional circRNAs in OS. Hsa_circ_0074834 facilitates osteogenesis-angiogenesis coupling in BMSCs via sequestering miR-942-5p^17^. Circ-RFWD2 and circ-INO80 contribute to HELL-1-caused osteogenesis in human adipose-derived stem cells^18^.

Circ-DAB1 is documented in the GEO dataset (GSE115196) deposited by Zheng Y et al.^19^ as one of the upregulated circRNAs in BMSCs during osteogenic differentiation at day 7 versus day 0. However, detailed function of circ-DAB1 in OS is never investigated. We screened the differentially expressed circRNAs in BMSCs during OS by qRT-PCR based on GSE115196 data, and found that circ-DAB1 was the most significantly upregulated one. Therefore, we planned to explore whether and how circ-DAB1 functioned in the osteogenic differentiation of human BMSCs.

## Materials and methods

### Cell culture and treatment

Human bone marrow stem cells (BMSCs; Shanghai Institutes for Biological Sciences, Shanghai, China) were preserved in the α-MEM (Sigma-Aldrich, Saint-Louis, Missouri) with addition of the 1% antibiotics and 10% fetal bovine serum (FBS) at 37°C with 95% air and 5% carbon dioxide. To induce osteogenic differentiation, osteogenic medium was applied using α-MEM added with 1% antibiotics, 10% FBS, 0.2 mM ascorbic acid, 10 mM β-glycerophosphate, and 100 nM dexamethasone. For treating cells, 3 U/μg of RNase R was procured from Epicentre Technologies (Madison, WI).

### Alkaline phosphatase staining

Alkaline phosphatase (ALP) staining analysis was implemented in accordance with the protocol of NBT/BCIP kit (CoWin Biotech, Beijing, China). BMSCs were maintained in the osteogenic medium for 7, 14, and 21 days in 24-well plates, followed by being fixed and incubated with staining reagent for 20 min in the dark.

### Mineralization assay

For detecting calcium deposition in extracellular matrix, BMSCs were subjected to 7, 14, and 21 days of incubation with osteogenic medium in 24-well plates. After fixation, calcified nodules were treated with the 0.1% Alizarin red S solution (Sigma-Aldrich) at PH 4.2.

### RNA extraction and qRT-PCR

Total RNA sample from BMSCs were extracted by TRIzol RNA reagent (Invitrogen, Carlsbad, CA), and 1 μg of RNA sample was prepared for reverse-transcription into cDNA using PrimeScriptRT kit (TaKaRa, Shiga, Japan). qRT-PCR procedure was operated on Applied Biosystems 7500 Real-time Fast PCR System with SYBR Green PCR Mastermix (Applied Biosystems, Foster City, CA). Gene expression level was standardized to GAPDH or U6, according to the 2^−∆∆Ct^ equation.

### Western blot

Total protein sample from BMSCs was isolated by RIPA lysis buffer and protease inhibitor, and sample was treated with electrophoresis for 70 min on 10% SDS-PAGE. After electroblotting to PVDF membranes (Millipore, Bellerica, MA), sample was incubated with 5% nonfat milk, prior to incubation with primary antibodies (Abcam, Cambridge, MA) against ALP (1:500, ab67228), RUNX2 (1:1000, ab23981), OSX (1:500, ab22552), OCN (1:500, ab93876), COL1A1 (1:1000, ab34710), RBPJ (1:1000, ab180588) and control (GAPDH, 1:1000, ab181602), as well as HRP-tagged secondary antibody (1:5000, ab175733). Protein levels were monitored by Image Quant LAS 4000 (GE Healthcare, Milwaukee, MI).

### Northern blot

Isolation of RNAs from BMSCs was achieved by TRIZOL (Invitrogen). Probes of circ-DAB1, linear DAB1 and GAPDH for northern blot were obtained using the Biotin RNA labeling mix (Roche). RNA samples were divided by electrophoresis, transferred to the Nitrocellulose (NC) membranes, and then were incubated with hydration buffer added with probes. Detection of RNA signal was conducted by Chemiluminescent Nucleic Acid Detection Module (Thermo Scientific).

### Agarose gel electrophoresis

The genomic DNA (gDNA) from BMSCs was extracted by DNeasy Blood & Tissue Kit (Qiagen, Hilden, Germany) in light of the standard method. Total RNA from BMSCs were extracted by TRIzol kit. Both gDNA and cDNA were treated with 1% agarose gels to detect their PCR products.

### RNA FISH assays

The specific FISH probe for circ-DAB1 was procured from RiboBio (Guangzhou, China) for incubation with cultured BMSCs in hybridization solution. After nuclear counterstaining with DAPI (Beyotime, Shanghai, China), cells were imaged under fluorescence microscope (Olympus Corp., Tokyo, Japan).

### Subcellular fractionation

In all, 1 × 10^6^ cultured BMSCs were rinsed in phosphate-buffered saline (PBS), and then placed into the cell fractionation buffer and cell disruption buffer in sequence. The fractions of cell cytoplasm or cell nucleus were individually separated in line with the manual of PARIS™ Kit (Invitrogen). Expression levels of circ-DAB1, U6, and GAPDH were analyzed by qRT-PCR.

### Transfection

Confluent BMSCs were prepared in the 6-well plates for 48 h with transfection plasmids using Lipofectamine2000 (Invitrogen). The short hairpin RNAs (shRNAs) against circ-DAB1 (sh-circ-DAB1#1: 5′-CCGGATATTTGGGCCTCTGGAGACACTCGAGTGTCTCCAGAGGCCCAATATTTTTTG-3′, sh-circ-DAB1#2: 5′-CCGGTTTGGGCCTCTGGAGACAGGTCTCGAGACCTGTCTCCAGAGGCCCAAATTTTTG-3′), RBPJ (sh-RBPJ#1: 5′-CCGGGCAGACTCATTGGGCTACATTCTCGAGAATGTAGCCCAATGAGTCTGCTTTTTG-3′, sh-RBPJ#2: 5′-CCGGCCAGTGACTTTGGTCCGAAATCTCGAGATTTCGGACCAAAGTCACTGGTTTTTG-3′), DAB1 (sh-DAB1: 5′-CCGGGTAAGTGCTGTGACCCAATTACTCGAGTAATTGGGTCACAGCACTTACTTTTTTG-3′) and the negative control (NC) shRNAs (sh-NC) were synthesized at RiboBio for gene silencing. The pcDNA3.1/circ-DAB1 and empty pcDNA3.1(+) circRNA mini vector, pcDNA3.1/RBPJ and empty pcDNA3.1 vector, miR-1270 mimic, miR-944 mimic, and NC mimic all were produced by Genepharma (Shanghai, China) for gene overexpression.

### Cell counting kit-8

The cultured BMSCs were reaped and seeded into 96-well plates (1 × 10^4^/well), then incubated with 10 μL of CCK8 solution (Beyotime) to measure cell viability. After 2 h, absorbance at 450 nm was recorded via microplate reader.

### Dual-luciferase reporter assay

For analyzing promoter, the DAB1 or RBPJ promoters were separately amplified by PCR for inserting into the pGL3 luciferase vectors (Promega, Madison, WI). BMSCs in 96-well plates (8 × 10^3^/well) were co-transfected with promoter vectors and pcDNA3.1/circ-DAB1 or control vector (NC). Besides, the wild-type (WT) and mutant (MUT) interacting sites for miR-1270 or miR-944 within RBPJ 3′-UTR or circ-DAB1 sequence were inserted into the pmirGLO luciferase vectors (Promega) to construct WT/MUT-RBPJ and WT/MUT-circ-DAB1 reporter vectors, and then indicated recombinant plasmids were co-transfected into 293T cells with NC mimic, miR-1270 mimic, miR-944 mimic, or miR-1270 mimic + miR-944 mimic, as needed. At length, the Dual-Luciferase Reporter Assay System (Promega) was prepared for detecting luciferase vectors at 48 h post-transfection.

### RNA immunoprecipitation (RIP)

In all, 1 × 10^7^ BMSCs after lysing in RNA immunoprecipitation (RIP) lysis buffer were collected for RIP assay with anti-RBPJ, anti-Ago2, or NC anti-IgG antibody. Following collecting the precipitates by magnetic beads, qRT-PCR was conducted for analyzing RNA enrichment.

### Pull down analyses

For RNA pull down, the protein extracts from BMSCs was utilized for mixing with 50 pmol of Bio-circ-DAB1 probe or No-biotin-circ-DAB1 probe as NC, and beads. Western blot and qRT-PCR were followed. For DNA pull down, the biotin-labeled promoter probe after PCR amplification was bound with beads for 4 h, with no biotin-probe as NC. After overnight incubation with cell lysates, complex was analyzed by western blot and qRT-PCR.

### Chromatin immunoprecipitation

The cross-linked chromatin was broken for Chromatin immunoprecipitation (ChIP) assay into 200–1000-bp fragments, then immunoprecipitated with 2 μg of anti-RBPJ or NC anti-IgG antibody. After the addition of beads, the collected chromatin was purified for qRT-PCR.

### Statistical analysis

Bio-triple repeats were implemented in all assays and results were shown as the mean ± SD. Data analyses were processed through ANOVA or *t* test, by employing PRISM 7 (GraphPad, San Diego, CA), with statistical significance was set as *p* < 0.05.

## Results

### BMSC osteoblast differentiation is induced

After BMSCs were cultured in osteogenic medium for 7, 14, and 21 days, we detected osteogenic differentiation of BMSCs by ALP and ARS staining. Pictures from ALP staining suggested that the ALP activity gradually increased in BMSCs cultured at day 7, 14, and 21 versus day 0 (Fig. S1A). ARS staining suggested that calcified nodules in BMSCs increased gradually after 7, 14, and 21 days of culturing (Fig. S1A). Also, the mRNA and protein expressions of five osteogenic markers (ALP, COL1A1, RUNX2, OSX, and OCN) were elevated at day 7, 14, and 21 of culturing (Fig. S1B, C). Above data confirmed that osteogenic differentiation is induced in BMSCs treated with the osteogenic medium.

### Circ-DAB1 is upregulated during BMSC osteogenic differentiation

**Fig. 1:**
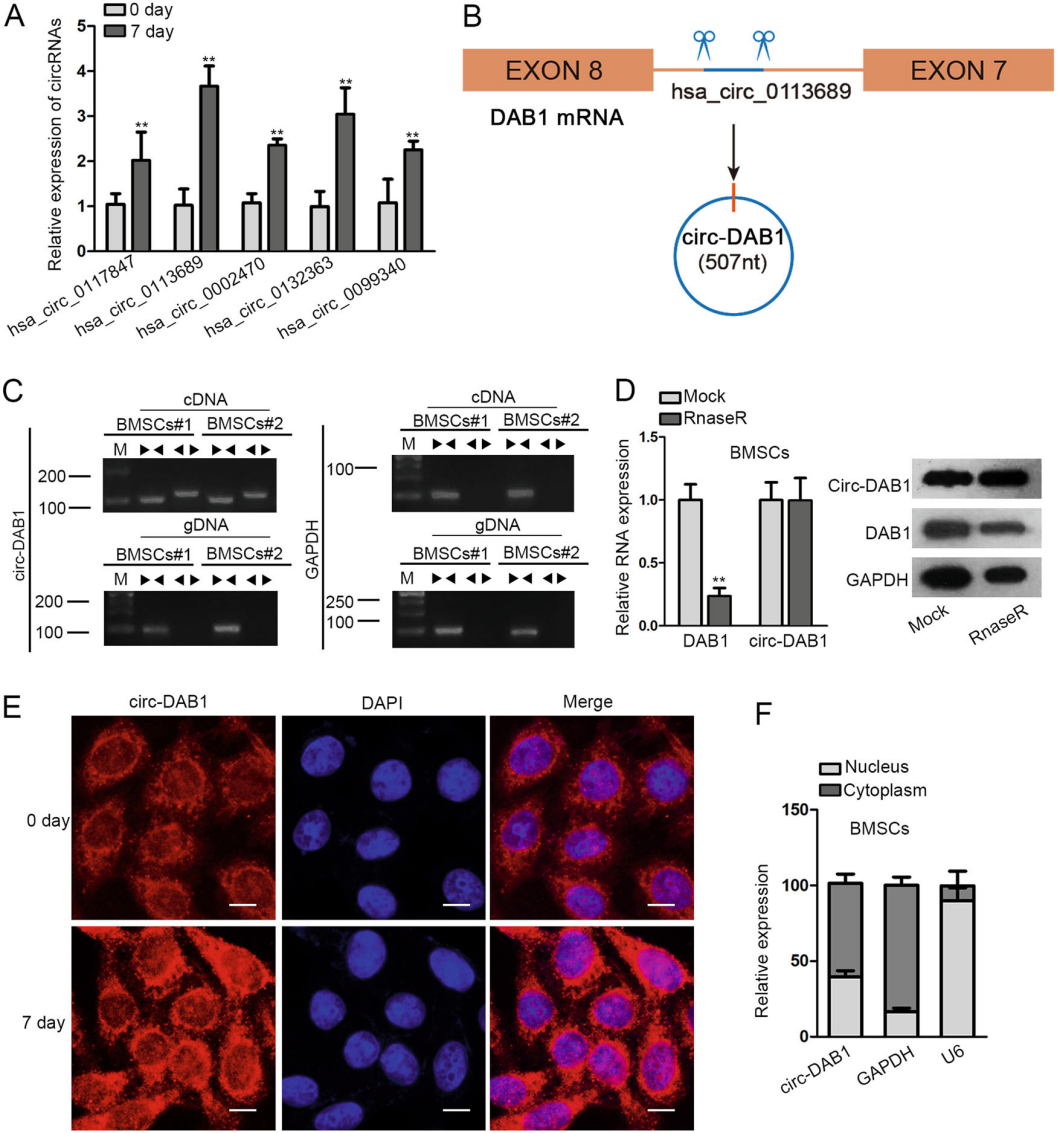
Circ-DAB1 was upregulated during BMSC osteogenic differentiation. **a** qRT-PCR validated the five most upregulated circRNAs in BMSCs during osteogenic differentiation at day 7 day versus day 0. **b** Schematic picture of the back-splicing of circ-DAB1 from DAB1. **c** Agarose gel electrophoresis corroborated the existence of circ-DAB1 in PCR products of divergent primers in cDNA but not gDNA in BMSCs. **d** qRT-PCR and northern blot of circ-DAB1, linear DAB1 mRNA, and GAPDH in BMSCs treated without or with RNase R. **e**, **f** FISH and subcellular fractionation detected the distribution of circ-DAB1. Scale bar: 30 μm. **P < 0.01. Experiments conducted with three biological repeats.

To screen out the circRNAs related to ostrogenesis, we referred to GSE115196 datasets, and obtained 240 upregulated circRNAs (*P* < 0.01, LogFC > 2) during osteogenic differentiation in BMSCs. qRT-PCR data showed that among these 240 circRNAs, 5 (hsa_circ_0117847, hsa_circ_0113689, hsa_circ_0002470, hsa_circ_0132363, and hsa_circ_0099340) were upregulated the most significantly in BMSC at day 7 in osteogenic medium, and the top-1 was circ-DAB1 (hsa_circ_0113689) (Fig. 1a). Thus, we suggested that circ-DAB1 was closely related to OS of BMSCs. As presented Fig. 1b circ-DAB1 was a 507-nt-long circRNA back-spliced from DAB1 between exon 8 and exon 8. To rule out genomic rearrangements and trans-splicing, the convergent primers were utilized to amplify DAB1 mRNA and divergent primers for circ-DAB1 amplification. Employing cDNA and gDNA from BMSCs as templates, we observed that circ-DAB1 was amplified with divergent primers from cDNA but not from genomic DNA (Fig. 1c). As widely known, circRNA is more resistant to RNase R degradation compared with mRNA^20^. Expectedly, qRT-PCR and northern blot data confirmed that RNase R significantly degraded DAB1 mRNA but not act circ-DAB1 (Fig. 1d). FISH staining showed more distribution of circ-DAB1 in cytoplasm than in nucleus in BMSCs (Fig. 1e), and subcellular fractionation also confirmed larger proportion of circ-DAB1 in cytoplasm (Fig. 1f). These findings implied that circ-DAB1 is a bona fide circRNA upregulated in BMSCs during osteogenic differentiation in vitro.

### Circ-DAB1 facilitates BMSC proliferation and osteogenic differentiation

**Fig. 2:**
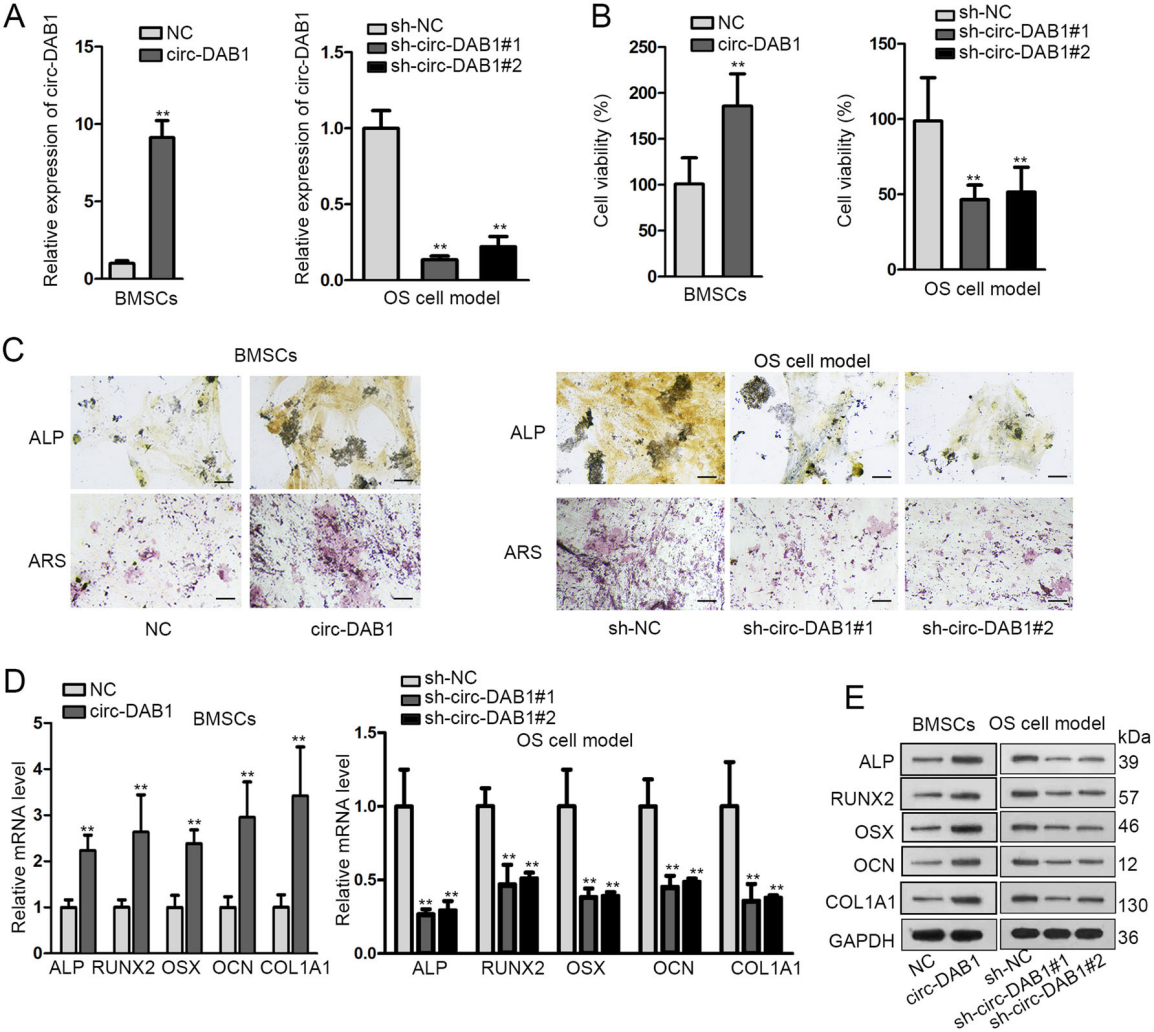
Circ-DAB1 facilitated BMSC proliferation and osteogenic differentiation. **a** qRT-PCR of circ-DAB1 level in untreated BMSCs (left) or BMSCs cultured for 7 days in osteogenic media (right) under the transfection of pcDNA3.1(+)/circ-DAB1 (circ-DAB1 group) versus vector (NC group) or sh-circ-DAB1#1/2 versus sh-NC. **b** CCK-8 analysis measured the proliferation of BMSCs or OS cell model following indicated transfections. **c** ALP and ARS staining in BMSCs or OS cell model following specific transfections. Scale bar: 100 μm. **d**, **e** qRT-PCR detection and western blots of the mRNA and protein of osteogenic markers in BMSCs or OS cell model following specific transfections. **P < 0.01. Experiments conducted with three biological repeats.

Then, we probed whether circ-DAB1 impacted osteogenic differentiation of BMSC. First of all, we overexpressed circ-DAB1 in untreated BMSCs and knocked down circ-DAB1 in OS cell model (BMSCs undergoing osteogenic differentiation for 7 days) (Fig. 2a). Using cell counting kit-8 (CCK-8) kit, we noted that viability of untreated BMSCs increased upon circ-DAB1 overexpression. In the meantime, circ-DAB1 deficiency reduced viability of osteogenic differentiated BMSCs (Fig. 2b). Besides, ALP and ARS staining indicated that circ-DAB1 overexpression increased ALP activity and facilitated the formation of calcified nodules in untreated BMSCs, whereas circ-DAB1 knockdown exerted opposite impacts in differentiated BMSCs (Figs. 2c and S2A). Moreover, upregulating circ-DAB1 increased the mRNA and protein expressions of osteogenic markers in BMSCs, and on the contrary, knocking down circ-DAB1 reduced mRNA and protein expressions of osteogenic genes in OS cell model (Fig. 2d, e). Altogether, circ-DAB1 can facilitate BMSC proliferation and osteogenic differentiation.

### Circ-DAB1 positively regulates DAB1 via inducing RBPJ

**Fig. 3:**
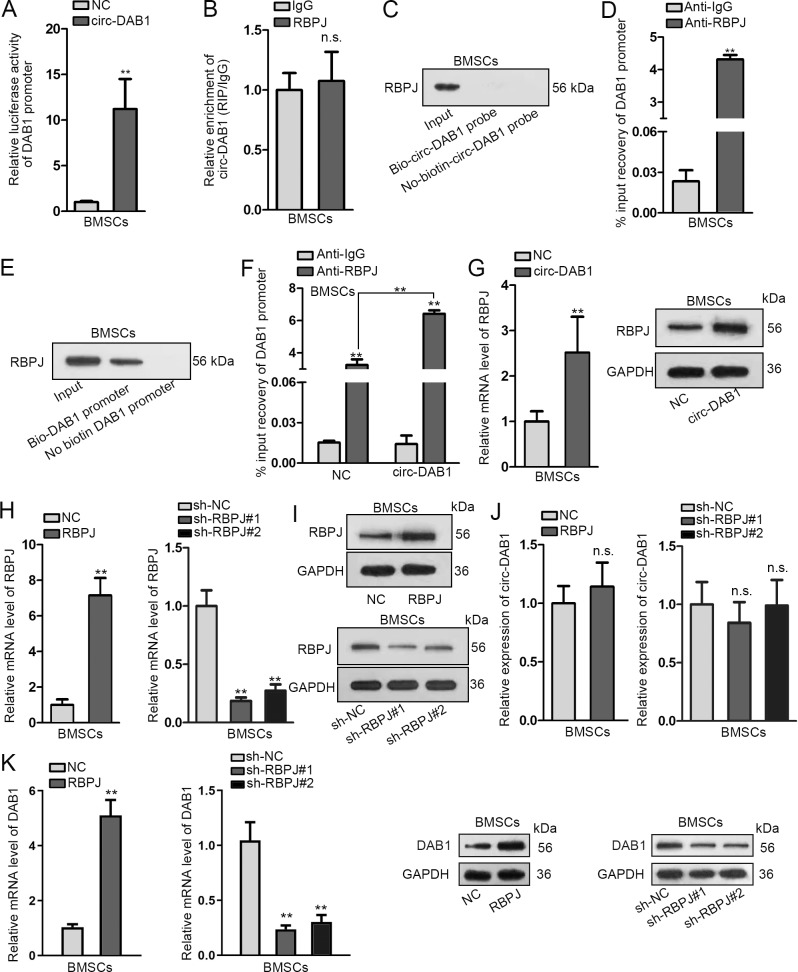
Circ-DAB1 positively regulated DAB1 via inducing RBPJ. **a** Luciferase activity of DAB1 promoter in response to circ-DAB1-overexpressing plasmids. **b**, **c** The interaction between circ-DAB1 and RBPJ was probed with RIP and RNA pull-down assays. **d**, **e** ChIP and DNA pull-down assays assessed the interaction between DAB1 promoter and RBPJ. **f** qRT-PCR analysis of the enrichment of DAB1 promoter in RBPJ ChIP products in BMSCs under circ-DAB1 overexpression. **g** qRT-PCR and western blots of RBPJ mRNA and protein levels in BMSCs with circ-DAB1 overexpression. **h**, **i** qRT-PCR and western blot of RBPJ level in BMSCs transfected with pcDNA3.1/RBPJ (RBPJ group) versus pcDNA3.1 (NC group) or with sh-RBPJ#1/2 versus sh-NC. **j** qRT-PCR of circ-DAB1 expression in BMSCs with RBPJ overexpression or knockdown. **k** qRT-PCR and western blot of DAB1 level in BMSCs with RBPJ overexpression or knockdown. **P < 0.01. n.s. indicated no significance. Experiments conducted with three biological repeats.

CircRNAs are supported to present high potential to regulate their associated genes^21,22^. Prior works demonstrated that DAB1, the host gene of circ-DAB1, could accelerate chondrogenesis which is another class of differentiation contributing to bone formation^23^. Thus, we wondered whether that circ-DAB1 could regulate DAB1 in osteogenic differentiation. Luciferase reporter assay presented that the luciferase activity of DAB1 promoter increased upon circ-DAB1 overexpression, hinting that circ-DAB1 positively affected DAB1 transcription (Fig. 3a). As formerly reported, DAB1 could be transcriptionally promoted in colorectal cancer via RBPJ, an important transcriptional factor of NOTCH pathway^24^. Hence, we tested whether nuclear circ-DAB1 transcriptionally initiated DAB1 expression via interacting with RBPJ. Surprisingly, circ-DAB1 exhibited no enrichment in the RIP products of RPBJ (Fig. 3b). Consistently, biotin-labeled circ-DAB1 (Bio-circ-DAB1 probe) did not pull down RBPJ protein (Fig. 3c). Based on these data, we suggested that circ-DAB1 could not directly interact with RBPJ. However, we observed through ChIP assay that DAB1 promoter was enriched RBPJ-interacting complexes (Fig. 3d). Also, DNA pull-down assay proved that RBPJ was pulled down by Bio-DAB1 promoter probe (Fig. 3e), confirming the interaction between RBPJ and DAB1 promoter. Interestingly, ChIP assay illustrated that that overexpression of circ-DAB1 increased DAB1 promoter enrichment in RBPJ-interacting complexes (Fig. 3f), indicating that circ-DAB1 increased the interplay between DAB1 promoter and RBPJ. Then, we found that overexpressing circ-DAB1 elevated the expression of RBPJ mRNA and protein (Fig. 3g). Meanwhile, to test the impact of RBPJ on DAB1 and circ-DAB1 expressions, we overexpressed or knocked down RBPJ in BMSCs (Fig. 3h, i). Consequently, circ-DAB1 expression was not altered by RBPJ overexpression or suppression (Fig. 3j), but DAB1 mRNA and protein increased under RBPJ overexpression and declined under RBPJ knockdown in BMSCs (Fig. 3k). Taken together, circ-DAB1 induces DAB1 expression via the upregulating RBPJ expression.

### Circ-DAB1 up-regulates RBPJ expression by sponging miR-1270 and miR-944

**Fig. 4:**
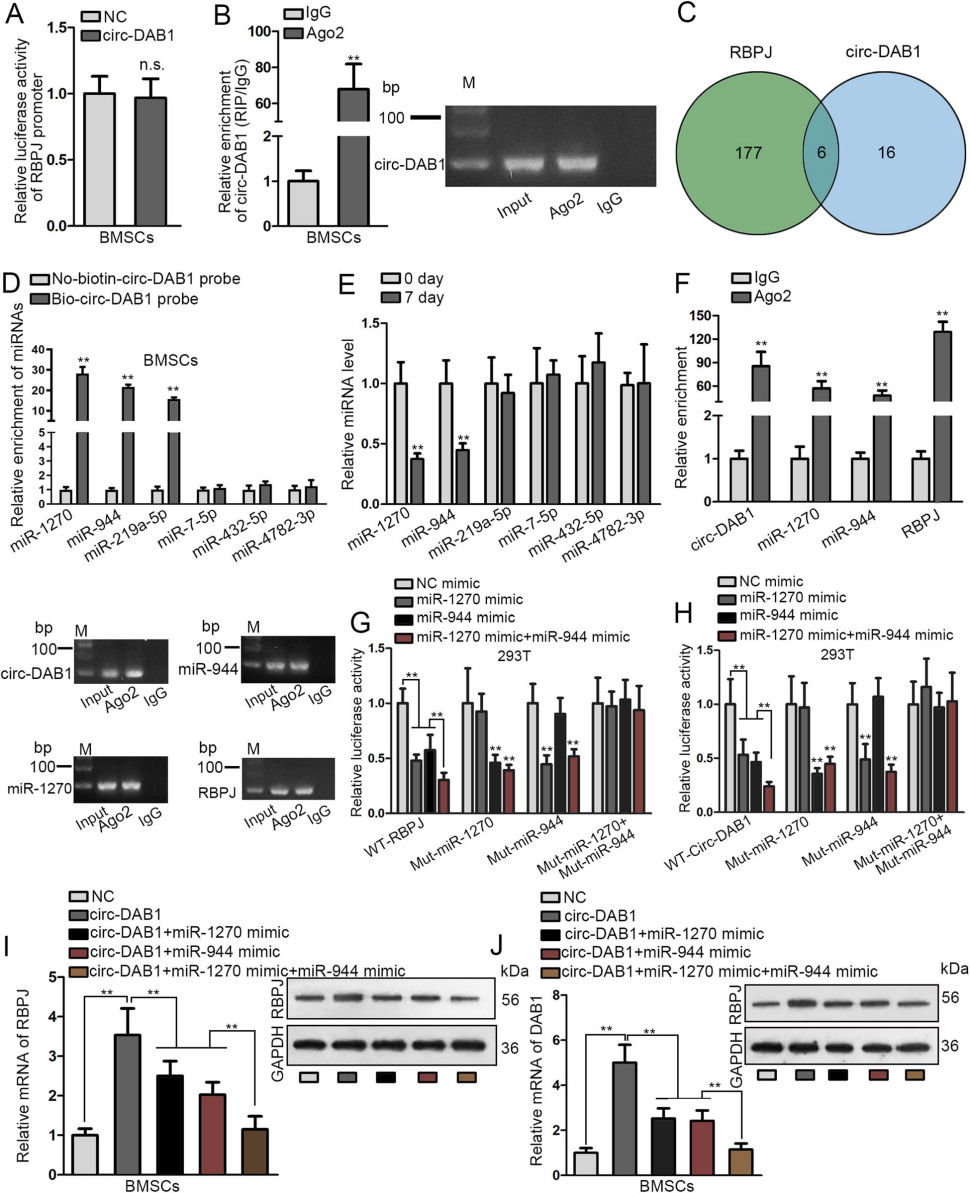
Circ-DAB1 upregulated RBPJ by sponging miR-1270 and miR-944. **a** Luciferase activity of RBPJ promoter in response to circ-DAB1-overexpressing vectors. **b** RIP assay monitored the enrichment of circ-DAB1 on the beads conjugated with Ago2 antibody. **c** StarBase predicted the miRNAs interacting with both circ-DAB1 and RBPJ. **d** RNA pull-down assay testified the enrichment of six miRNAs using No-biotin-circ-DAB1 probe and Bio-circ-DAB1 probe. **e** qRT-PCR analysis of six miRNA expressions in BMSCs following osteogenic medium cultivation. **f** Ago2-RIP verified the abundance of circ-DAB1, miR-1270, miR-944, and RBPJ in RISC. **g**, **h** 293T were transfected with NC mimic, miR-1270 mimic, miR-944 mimic, and miR-1270 mimic+miR-944 mimic; the luciferase activity of RBPJ and circ-DAB1 were evaluated. **i**, **j** The mRNA and protein levels of RBPJ or DAB1 were assessed in BMSCs transfected with the indicated plasmids. **P < 0.01. n.s. indicated no significance. Experiments conducted with three biological repeats.

Subsequently, we proceeded to figure out the how circ-DAB1 induced RBPJ expression. We found that circ-DAB1 overexpression failed to alter RBPJ promoter activity (Fig. 4a). Since we previously validated that circ-DAB1 was distributed more in the cytoplasm, we hypothesized that circ-DAB1 regulated RBPJ post-transcriptionally. We then found that circ-DAB1 was precipitated by anti-Ago2, suggesting its abundance in RNA-induced silencing complex (RISC) (Fig. 4b). Hence, we speculated that circ-DAB1 mediated ceRNA network in BMSCs. Through starBase (http://starbase.sysu.edu.cn/index.php), 6 miRNAs (miR-4782-3p, miR-432-5p, miR-7-5p, miR-219a-5p, miR-944, and miR-1270) were predicted to bind to both circ-DAB1 and RBPJ mRNA (Fig. 4c). qRT-PCR proved the significant enrichment of miR-1270, miR-944, and miR-219a-5p in the pulldown of Bio-circ-DAB1 probe (Fig. 4d). Besides, we measured the expressions of 6 miRNAs in BMSCs following incubation in osteogenic medium. qRT-PCR data illustrated that miR-1270 and miR-944 declined in BMSCs during osteogenic differentiation (Fig. 4e). Thus, we speculated that circ-DAB1 targeted miR-1270 and miR-944 in BMSCs to regulate RBPJ.

RIP assay indicated that circ-DAB1, miR-1270, miR-944, and RBPJ were all markedly enriched in Ago2 RIP (Fig. 4f). As shown in Fig. S3A, overexpressing miR-1270 or miR-944 could not regulate circ-DAB1 level. Also, overexpressing circ-DAB1 failed to alter miR-1270 and miR-944 levels (Fig. S3B). Luciferase activity of WT-RBPJ declined by miR-1270 mimic or miR-944 mimic in 293T cells, and such decline was strengthened by co-transfection of miR-1270 mimic and miR-944 mimic (Fig. 4g). Similarly, WT-circ-DAB1 luciferase activity was repressed by miR-1270 mimic or miR-944 mimic, and such repression was enhanced under the co-transfection of miR-1270 mimic and miR-944 mimic (Fig. 4h). Moreover, qRT-PCR and western blot proved that overexpressing circ-DAB1 elevated RBPJ and DAB1 expression, and such effect was partially reversed by respectively overexpressing miR-1270 or miR-944, and fully reversed by joint overexpression of miR-1270 and miR-944 (Fig. 4i, j). Overall, circ-DAB1 promotes RBPJ expression by competitively binding to miR-1270 and miR-944.

### Depletion of RBPJ or DAB1 abrogated the effects of circ-DAB1 in BMSC osteogenic differentiation

**Fig. 5:**
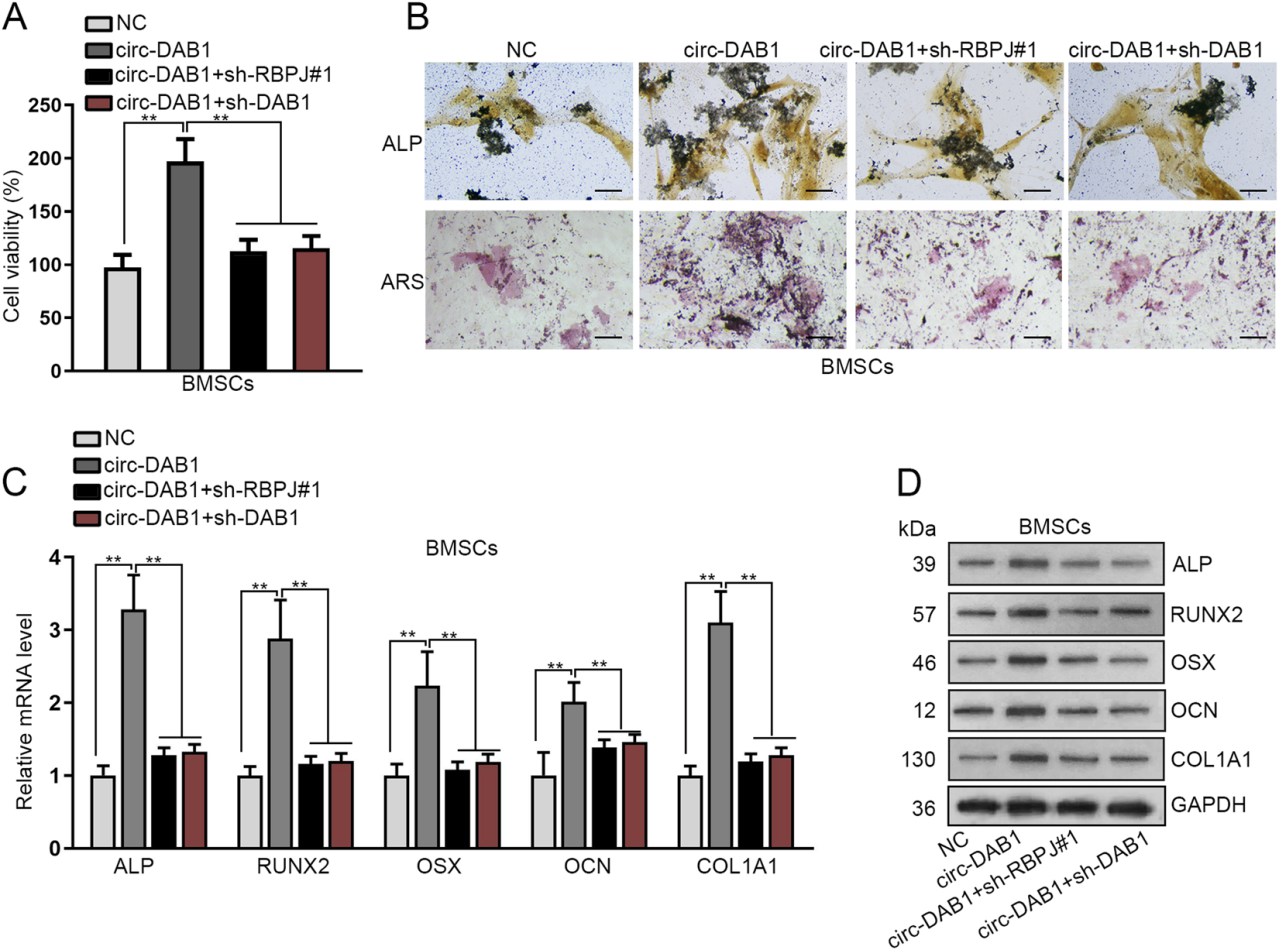
Depletion of RBPJ or DAB1 abrogated the effects of circ-DAB1 in BMSC osteogenic differentiation. **a** BMSCs were transfected with NC, circ-DAB1, circ-DAB1 + sh-RBPJ, or circ-DAB1 + sh-DAB1; CCK-8 detected the proliferation of BMSCs. **b** ALP/ARS staining of BMSCs following specific transfections. Scale bar: 100 μm (**c**, **d**) qRT-PCR and western blot examined the expressions of osteogenic markers in BMSCs. **P < 0.01. Experiments conducted with three biological repeats.

Rescue experiments were carried out to functionally confirm circ-DAB1/RBPJ/DAB1 axis in BMSCs. As described in CCK-8 assay, the increased cell proliferation of BMSCs caused by circ-DAB1 overexpression could be abrogated by RBPJ or DAB1 down-regulation (Fig. 5a). Furthermore, the promoting effects of circ-DAB1 overexpression on ALP activity and calcified nodules were reversed by the knockdown of RBPJ or DAB1 (Figs. 5b and S4A). Meanwhile, as exhibited by qRT-PCR and western blot, circ-DAB1 enhancement-caused upregulation of osteogenic markers was neutralized by sh-RBPJ or sh-DAB1, (Fig. 5c, d). Conclusively, circ-DAB1 facilitates BMSC osteoblast differentiation RBPJ/DAB1 axis.

## Discussion

Volumes of studies highlighted coding and noncoding RNAs as the transcriptional or post-transcriptional regulators of genes in osteogenic differentiation of BMSCs^6,7^. CircRNAs are a class of noncoding RNAs that are expressed under a stable and cell-type-specific condition^14,25^ They regulate diverse cellular behaviors^9^, and are considered as potential diagnostic and therapeutic biomarker in various types of diseases, such as cancer and bone disorders^26–28^. Some circRNAs are identified as participants in osteogenesis such as hsa_circ_0074834^17^, circ-RFWD2 and circ-INO80^18^. Based on GEO data, we obtained 240 circRNAs reported to be upregulated in BMSCs during osteogenic differentiation. Our qRT-PCR data showed that circ-DAB1 was the most upregulated circRNA in BMSCs cultured in osteogenic medium at day 7, confirming the association between circ-DAB1 and osteogenesis. We first demonstrated the function that overexpressing circ-DAB1 enhanced cell proliferation and osteogenic differentiation of BMSCs, and silencing circ-DAB1 presented the reverse effect. This evidence proved that circ-DAB1 was a positive modulator for osteogenic differentiation of BMSCs.

Several reports indicate the potential of circRNAs to target host gene^21,22^. Herein, DAB1 is the host gene of circ-DAB1 and best known to regulate the development of neuron migration and lamination^29^. DAB1 was reported to be recruited by RBX2 to maintain retinal cell position^30^. More importantly, it was reported that DAB1 exerted promoting role in chondrogenesis, and chondrogenesis is also a type of differentiation contributing to bone formation^23^. Thus, we suggested that DAB1 potentially participate in osteogenesis as a circ-DAB1 target gene. We discovered that circ-DAB1 affected DAB1 transcription. Previously, DAB1 transcription was reported to be activated by RBPJ in colorectal cancer^24^. RBPJ is the essential transcription factor in Notch pathway and regulate receptor-ligand interactions to modify gene expression^31^. Also, Notch is a pathway supported to facilitate osteogenesis^32^. It has been reported that RBPJ-dependent Notch signals modulate the development of cartilage and bone^33^. Consistently, we first proved that RBPJ also activated DAB1 transcription in BMSCs. Interestingly, circ-DAB1 failed to interact with RBPJ, but can induce RBPJ expression, indicating that circ-DAB1 increased RBPJ-DAB1 promoter interaction by increasing RBPJ expression in BMSCs.

CeRNA network is a typical post-transcriptional regulatory mechanism that RNA transcripts, like circRNAs and lncRNAs, share the response elements of miRNAs and competitively bind to miRNAs to regulate target gene expressions^34^. miRNAs are largely reported in osteogenesis. For example, exosomal miR-23a-5p derived from osteoclast represses osteogenesis^35^, Mutant Runx2 modulates osteogenesis through miR-185-5p/Dlx2^36^. Herein, we identified miR-1270 and miR-944 as two targets for circ-DAB1 and showed that these 2 miRNAs were downregulated in BMSCs during osteogenic differentiation. MiR-1270 has been studied to be regulated by circRNA Cdr1as in bladder cancer^37^. Notably, miR-1270 was identified to be upregulated in patents with osteonecrosis of the femoral head who presented reduced osteogenesis^38^, indicating the link between miR-1270 and osteogenesis. miR-944 was supported as a tumor-suppressor gene in cancers such as colorectal cancer^39^ and cervical cancer^40^, but miR-944 in osteogenic differentiation was not uncovered until this study. We provide first evidence that both miR-1270 and miR-944 was downregulated in BMSCs during osteogenic differentiation and that circ-DAB1 interacted with circ-DAB1 miR-1270 and miR-944 to upregulate RBPJ and DAB1. Through restoration experiments, we observed that the knockdown of RBPJ or DAB1 partially counteracted the facilitative effect of circ-DAB1 overexpression in the proliferation and osteogenic differentiation of BMSCs, indicating that RBPJ/DAB1 was involved in the regulation of circ-DAB1 on BMSC osteogenic differentiation.

**Fig. 6:**
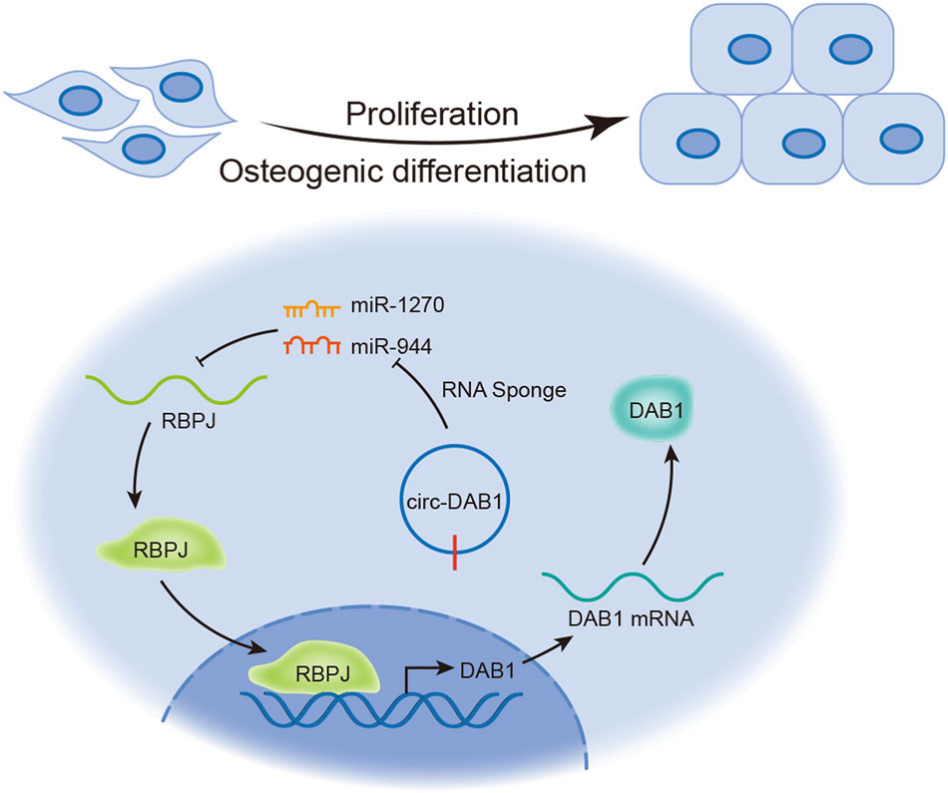
Mechanism diagram. Circ-DAB1 sponges miR-1270/miR-944 to upregulate RBPJ and RBPJ induced DAB1 transcription to upregulate DAB1, resulting in facilitated BMSC osteogenic differentiation.

In conclusion, circ-DAB1 upregulates DAB1 and promotes cell proliferation and osteogenic differentiation of BMSCs via miR-1270/miR-944/RBPJ signaling (Fig. 6), suggesting that circ-DAB1/RBPJ/DAB1 pathway could provide a meaningful revelation for exploring the underlying therapeutic strategies of osteogenic differentiation.

## Supplementary information

Supplementary figures legends

Figure S1

Figure S2

Figure S3

Figure S4
