# Supplementary material for: A circular RNA derived from DAB1 promotes cell proliferation and osteogenic differentiation of BMSCs via RBPJ/DAB1 axis
Source: Cell Death Dis. 2020 May 15;11(5):372. doi: 10.1038/s41419-020-2572-3 (PMC7229165; doi:10.1038/s41419-020-2572-3)
Supplement: Supplementary file 1 — Supplementary figures legends [file 41419_2020_2572_MOESM1_ESM.docx]

**Figure S1. BMSC osteoblast differentiation was induced.**

(A) Microscope captured the pictures of ALP and ARS staining in BMSCs cultured in osteogenic medium for 0, 7, 14, and 21 days. Scale bar: 100μm. (B-C) qRT-PCR and western blot data showed the mRNA and protein levels of five osteogenic markers in BMSCs cultured in osteogenic medium at day 0, 7, 14 and 21. ^**^P < 0.01. Experiments conducted in 3 biological repeats.

**Figure S2. ALP and ARS staining in BMSCs with circ-DAB1 overexpression or inhibition.**

(A) Panoramic scanning of ALP and ARS in BMSCs for Figure 2C. Experiments conducted in 3 biological repeats.

**Figure S3. Regulation between circ-DAB1 and miR-1270/miR-944.**

(A) qRT-PCR of circ-DAB1 level under miR-1270 and miR-944 overexpression in BMSCs. (B) qRT-PCR of miR-1270 and miR-944 expressions in BMSCs under circ-DAB1 overexpression. n.s. denoted no significance. Experiments conducted in 3 biological repeats.

**Figure S4. ALP and ARS staining in indicated BMSCs.**

(A) Panoramic scanning of ALP and ARS staining in BMSCs for Figure 5B. Experiments conducted in 3 biological repeats.
