# Supplementary figures and images for: A circular RNA derived from DAB1 promotes cell proliferation and osteogenic differentiation of BMSCs via RBPJ/DAB1 axis
Source: Cell Death Dis. 2020 May 15;11(5):372. doi: 10.1038/s41419-020-2572-3 (PMC7229165; doi:10.1038/s41419-020-2572-3)

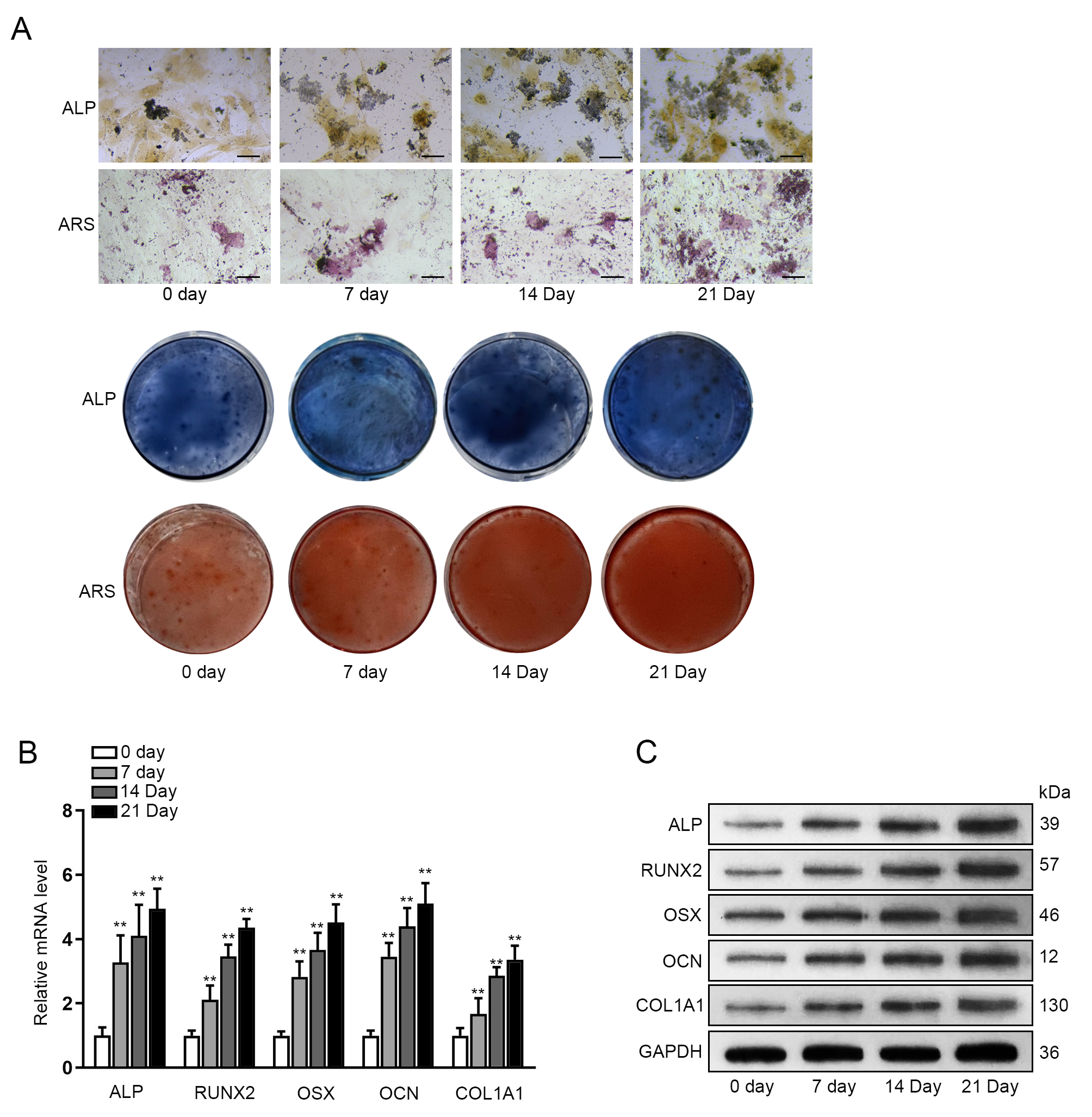

Supplement: Supplementary file 2 — Figure S1 [file 41419_2020_2572_MOESM2_ESM.tif]

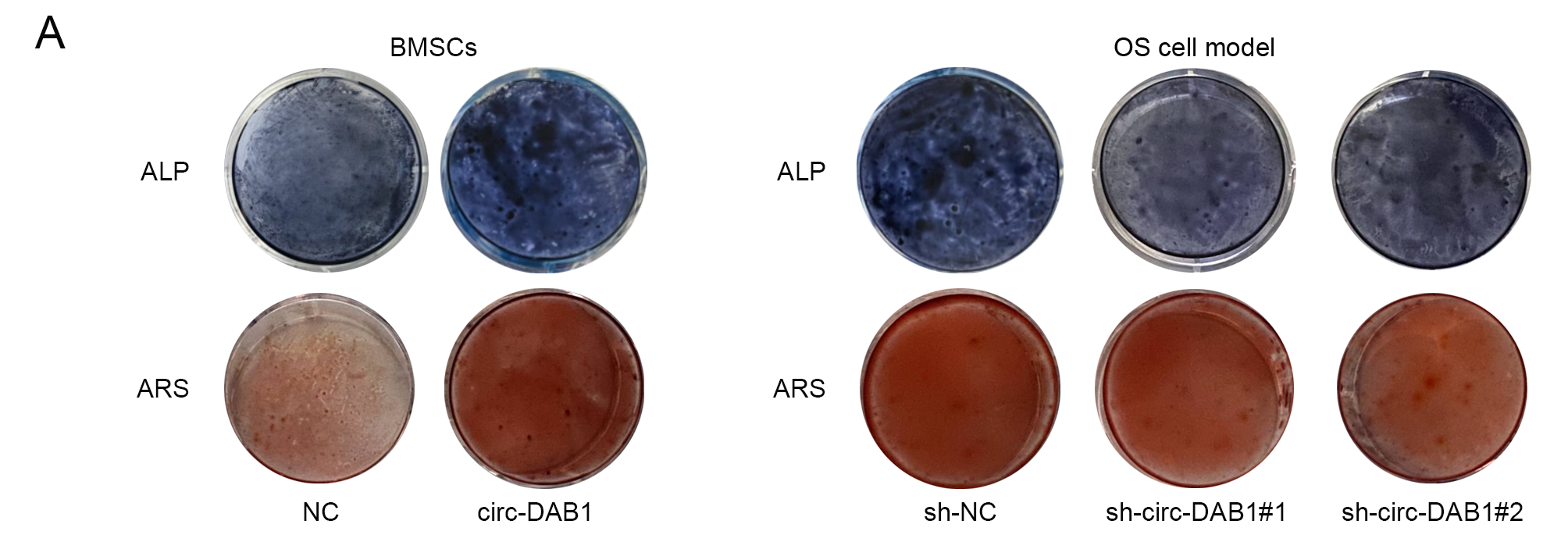

Supplement: Supplementary file 3 — Figure S2 [file 41419_2020_2572_MOESM3_ESM.tif]

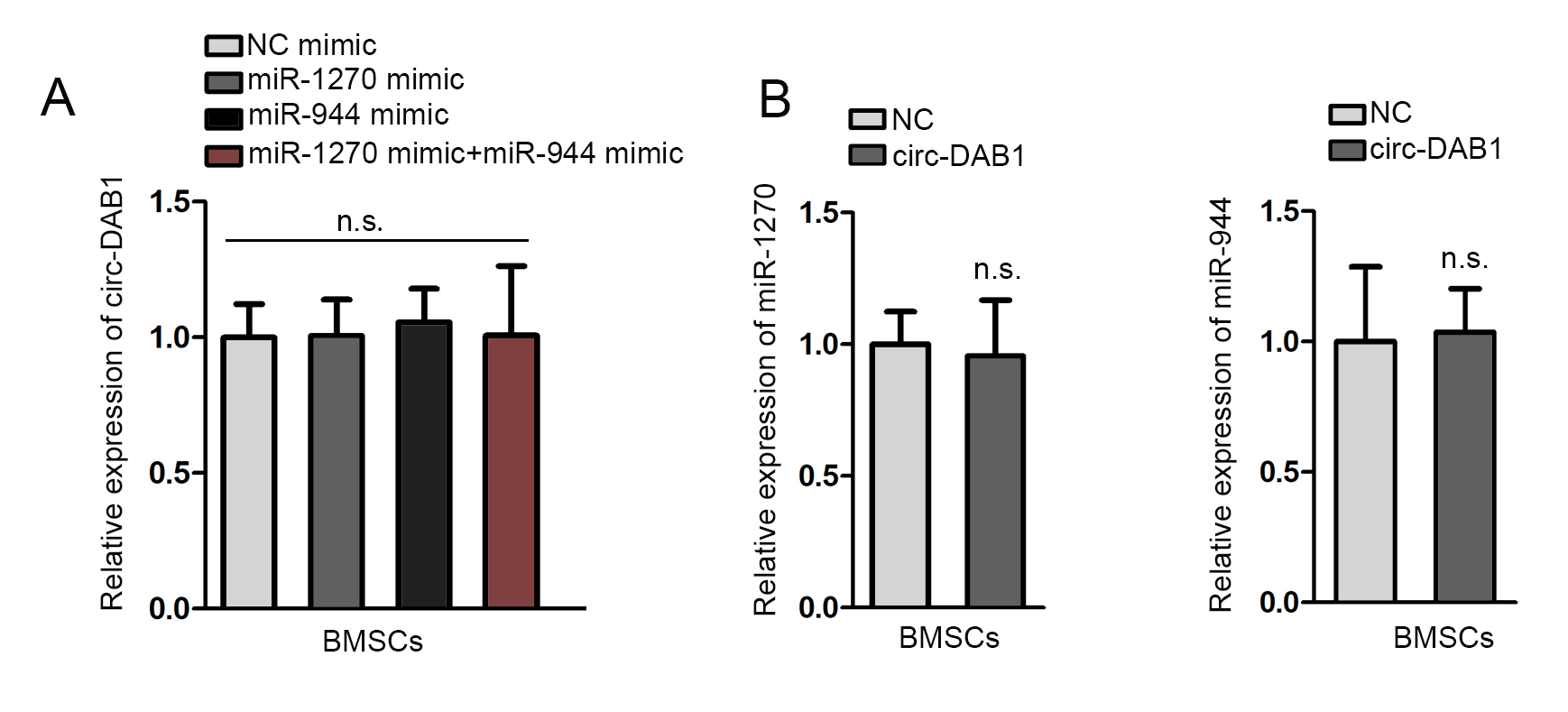

Supplement: Supplementary file 4 — Figure S3 [file 41419_2020_2572_MOESM4_ESM.tif]

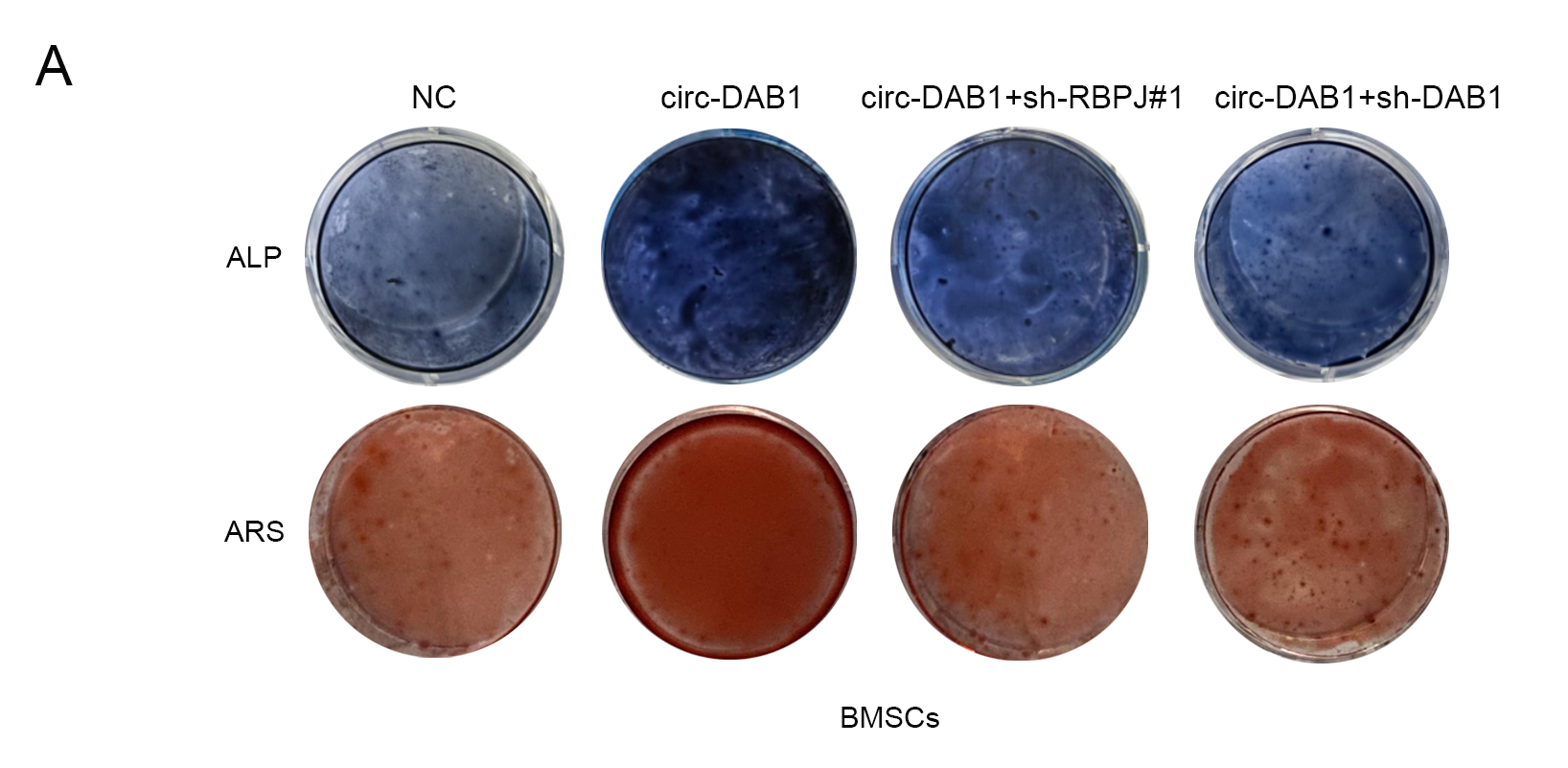

Supplement: Supplementary file 5 — Figure S4 [file 41419_2020_2572_MOESM5_ESM.tif]
